# A multivariate analysis on prognostic factors for lobular carcinoma of the breast

**DOI:** 10.1590/S1516-31802010000300004

**Published:** 2010-05-06

**Authors:** Hugo Fontan Köhler, Maria do Socorro Maciel, Juan Donoso Collins, Renato de Lima Rozenowicz, Mário Mourão

**Affiliations:** I MD. Former staff member, Department of Breast Surgery, Hospital A. C. Camargo, São Paulo, Brazil.; II MD, PhD. Attending physician, Department of Breast Surgery, Hospital A. C. Camargo, São Paulo, Brazil.; III MD. Attending physician, Department of Breast Surgery, Hospital A. C. Camargo, São Paulo, Brazil.; IV MD, PhD. Director, Department of Breast Surgery, Hospital A. C. Camargo, São Paulo, Brazil.

**Keywords:** Carcinoma, lobular, Prognosis, Lymph node excision, Drug therapy, Radiotherapy, Carcinoma lobular, Prognóstico, Excisão de linfonodo, Quimioterapia, Radioterapia

## Abstract

**CONTEXT AND OBJECTIVE:**

Lobular carcinoma is the second most common type of breast neoplasia and has unique clinical and pathological features. Our aim was to evaluate prognostic factors for this type of breast cancer.

**DESIGN AND SETTING:**

Retrospective study at a tertiary oncological institution.

**METHODS:**

162 patients diagnosed and treated between January 1985 and January 2002 were included. The inclusion criteria were: absence of previous treatment, histological diagnosis of lobular carcinoma, no previous history of breast cancer and minimum follow-up of 36 months.

**RESULTS:**

In univariate analysis, the following factors were statistically significant: clinical stage T (P = 0.0005), clinical stage N (P = 0.0014), neoadjuvant chemotherapy (P = 0.0008), primary tumor size (P < 0.0001), vascular invasion (P < 0.0001), lymphatic invasion (P = 0.0004), neural invasion (P = 0.0004), skin invasion (P < 0.0001), capsular transposition (P = 0.0008), lymph node ratio (P < 0.0001), estrogen receptor expression (P = 0.0186), progesterone receptor expression (P = 0.0286), pathological stage T (P < 0.0001), pathological stage N (P < 0.0001), adjuvant chemotherapy (P < 0.0001) and postoperative hormone therapy (P = 0.0367). After grouping the variables, multivariate analysis was performed. Presence of lymph node metastases, capsular transposition, lymph node ratio and postoperative hormone therapy remained significant.

**CONCLUSION:**

In this series, the most important prognostic factors for lobular carcinoma of the breast seemed to relate to lymph node status and presence of capsular transposition. Factors relating to axillary involvement, capsular transposition and hormone therapy were significant for survival.

## RESUMO

**CONTEXTO E OBJETIVO:**

O carcinoma lobular é o segundo tipo de neoplasia mais frequente na mama e tem características clínicas e patológicas próprias. Nosso objetivo foi avaliar fatores prognósticos para esse tipo de câncer de mama.

**TIPO DE ESTUDO E LOCAL:**

Estudo retrospectivo em instituição terciária oncológica.

**MÉTODOS:**

162 pacientes diagnosticadas e tratadas entre janeiro de 1985 e janeiro de 2002 foram incluídas. Os critérios de inclusão foram: ausência de tratamento prévio, diagnóstico histológico de carcinoma lobular, ausência de história prévia de câncer de mama e acompanhamento mínimo de 36 meses.

**RESULTADOS:**

Em análise univariada, os seguintes fatores foram estatisticamente significativos: estágio T clínico (P = 0,0005), estágio N clínico (P = 0,0014), quimioterapia neoadjuvante (P = 0,0008), tamanho do tumor primário (P < 0,0001), invasão vascular (P < 0,0001), invasão linfática (P = 0.0004), invasão neural (P = 0,0004), invasão de pele (P < 0,0001), transposição capsular (P = 0,0008), relação linfonodal (P < 0,0001), expressão de receptor estrogênico (P = 0,0168), expressão de receptor de progesterona (P = 0,0286), estágio T patológico (P < 0,0001), estágio N patológico (P < 0,0001), quimioterapia adjuvante (P < 0,0001) e hormonioterapia pós-operatória (P = 0.0367). Agrupando-se as variáveis, realizou-se análise multivariada. Presença de metástases linfonodais, transposição capsular, razão linfonodal e hormonioterapia pós-operatória permaneceram significantes.

**CONCLUSÃO:**

Nesta série, os fatores prognósticos mais importantes para carcinoma lobular de mama parecem ser aqueles relacionados com status linfonodal e presença de transposição capsular. Fatores relacionados ao comprometimento axilar, transposição capsular e terapia hormonal foram significativos para sobrevida.

## INTRODUCTION

Invasive lobular carcinoma (ILC) is the second most common type of breast cancer after invasive ductal carcinoma (IDC), accounting for 5-15% of all breast cancer cases.^1^ Its incidence rates increased from 1987 to 1999, and predominantly in postmenopausal women, in contrast to IDC rates, which remained largely constant throughout this period. The proportion of breast cancers with a lobular component increased from 9.5% in 1987 to 15.6% in 1999.^2^

The morphological features of lobular carcinoma differ from those of ductal carcinoma. ILC is characterized by small, round cells that are bland in appearance and have sparse cytoplasm. These cells infiltrate the stroma in single file and surround benign breast tissues in a targeted manner. Infiltration typically does not destroy anatomical structures or induce a substantial connective tissue response. By virtue of their distinctive growth pattern and biology, lobular carcinomas often fail to form distinct masses that can easily be diagnosed by palpation or mammography.^3,4^

There are clinical and pathological differences between ILC and IDC. A large retrospective study compared the clinical and biological features of 4,140 patients with ILC with those of 45,169 patients with IDC.^5^ Compared with IDC, ILC occurred significantly more frequently in older patients, was larger in size and was frequently more estrogen receptor and progesterone receptor-positive. Moreover, ILC had a lower S-phase fraction and tended to be diploid and HER-2, p53 and epidermal growth factor receptor-negative.^5-7^

Multivariate analysis has not identified any prognostic differences associated with ILC and IDC. The same standard prognostic factors (tumor size, axillary nodal status, hormone receptors, S-phase and age) are applicable both to lobular and to ductal carcinoma.^5^

## OBJECTIVE

The purpose of this paper was to report on a multivariate analysis of ILC using a single institution's experience, and to attempt to define prognostic factors for ILC that are not based on comparisons with IDC.

## PATIENTS AND METHODS

Patients with a histological diagnosis of ILC of the breast who were treated between January 1985 and January 2002 were selected for this study. All these patients were enrolled at a single private tertiary cancer center. The inclusion criteria were absence of previous treatment, confirmed histology with review at our institution, no previous history of breast cancer and minimum follow-up of 36 months after conclusion of treatment, except for patients who died due to treatment complications or other causes. The histological diagnosis included optical microscopy examination of samples from all the patients, while immunohistochemical studies were indicated at the pathologist's discretion. Patients with distant metastasis at diagnosis were excluded from this series. From these criteria, 162 patients were identified and included in this study.

The patients were staged in accordance with the 2002 TNM classification.^8^ The following data were collected: age, race, symptoms, previous gynecological history, clinical and pathological staging, treatment, pathological characteristics of the tumor, hormone receptor status of the primary tumor, presence of c-erb-B2, recurrence (if present) and status at last follow-up.

The statistical analysis was performed using the Statistical Package for the Social Sciences (SPSS) package, version 11 for MacOS X. P values greater than 0.05 were considered non-significant throughout the study. Initially, a univariate analysis was performed and the factors identified as significant were included in a multivariate analysis. This was performed using chunkwise testing methods with a backward elimination procedure. A set of logically related predictors of equal importance constituted the first chunk and backward elimination was applied. Then, respectively, new factors were added to the chunks and the backward elimination was reapplied, while significant factors were kept throughout the steps.^9^ Survival curves were constructed and compared using the Kaplan-Meier and Cox methods.

## RESULTS

All the patients were female. The age at diagnosis ranged from 28 to 87 years (mean of 55.89 years and median of 55.50 years). For statistical purposes, the patients were divided according to a cutoff point of 45 years: 36 patients (22.2%) were younger than 45 years and 126 (77.8%) were above the cutoff point. According to race, 134 were white and 28 were nonwhite.

In 142 patients (87.7%), clinical symptoms were present at the time of diagnosis, while in 20 (12.3%), only radiological findings were indicative of breast disease. Forty-five patients (27.8%) reported previous use of exogenous hormones as contraceptives (32 patients, 19.8%) or hormone replacement (13 patients, 8.0%). Thirty patients (18.5%) had a family history of breast cancer.

The patients were classified according to clinical stage, as T1a in two cases (1.2%), T1b in four cases (2.5%), T1c in 31 cases (19.1%), T2 in 82 cases (50.6%), T3 in 13 cases (8.0%) and T4b in 30 cases (18.5%). The axillary and supraclavicular nodes were staged as N0 in 90 cases (55.6%), N1 in 62 cases (38.3%) and N2 in 10 cases (6.2%). The pathological staging was pT1b in 10 cases (6.2%), pT1c in 26 patients (16.0%), pT2 in 78 cases (48.1%), pT3 in 29 cases (17.9%) and pT4b in 19 cases (11.7%). The number of retrieved nodes ranged from 7 to 63 (mean, 23.02 and median, 23.00 nodes), and the number of pathologically metastatic nodes ranged from 0 to 34 (mean, 5.23 and median, 0.50 nodes). From the pathological examination on the retrieved nodes, 82 patients (50.6%) were staged as pN0, 23 patients (14.2%) as pN1a, 24 patients (14.8%) as pN2a and 33 patients (20.4%) as pN3. The lymph node ratio was defined as the number of metastatic nodes divided by the number of dissected nodes and it had a mean value of 0.11 (standard deviation, SD, 0.07). Some patients were tested for hormone markers and prognostic factors. Estrogen receptor status was assessed in 154 patients (94.1%), progesterone receptor status in 72 patients (44.4%), p53 status in 42 patients (25.9%) and cerbB2 status in 43 patients (26.5%).

The treatment was based on the protocols used at the time of diagnosis. Preoperative chemotherapy was indicated for 13 patients (8.0%). Mastectomy was performed on 124 patients (76.5%), while breast-conserving surgery was performed on 38 patients (23.5%). In the postoperative setting, chemotherapy was administered for 87 patients (53.7%), radiotherapy for 100 patients (61.7%) and hormone therapy for 74 patients (45.7%). At the time of this study, trastuzumab was not used as routine chemotherapy, and no patients received this drug.

The duration of follow-up ranged from 1.45 to 147.78 months (mean, 75.87 and median, 56.53 months). There were six cases of local recurrence (3.7%), four of regional recurrence (2.5%) and 50 of distant recurrence (30.9%). At the last contact, 105 patients (64.8%) were alive and without evidence of active disease, one patient (0.6%) had active disease, 50 patients (30.9%) had died due to disease progression and six patients (6.7%) had died from other, unrelated causes.

Initially, univariate analysis was performed to enable identification of the factors that were significant for survival. The variables that showed significance in the analysis were: clinical stage T (P = 0.0005), clinical stage N (P = 0.0014), neoadjuvant chemotherapy (P = 0.0008), size of the primary tumor, using 3.50 centimeters as the cutoff point (P < 0.0001), vascular invasion (P < 0.0001), lymphatic invasion (P = 0.0004), neural invasion (P = 0.0004), skin invasion (P < 0.0001), capsular transposition (P = 0.0008), estrogen receptor (ER) expression (P = 0.0186), progesterone receptor (PgR) status (P = 0.0286), pathological stage T (P < 0.0001), pathological stage N (P < 0.0001), lymph node ratio (P < 0.0001), postoperative chemotherapy (P < 0.0001) and postoperative hormone therapy (P = 0.0367). The following factors were found to be nonsignificant: age group (P = 0.497), race (P = 0.8770), previous history of hormone therapy (P = 0.7825), previous family history (P = 0.0901), type of surgery (P = 0.0587), presence of desmoplasia (P = 0.2720), inflammatory infiltrate (P = 0.0567), comedocarcinoma (P = 0.3681), histological grading (P = 0.1800), bilateral disease (P = 0.285) and postoperative radiotherapy (P = 0.3037).

**Figure 1:**
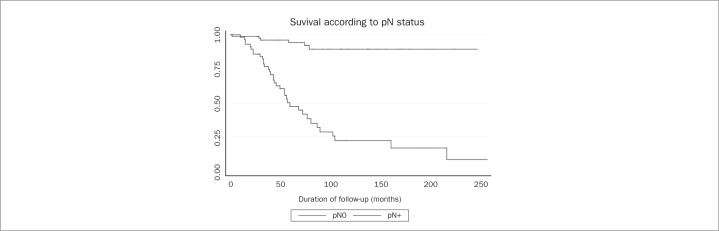
Overall survival rates for patients with and without metastatic axillary nodes. pN = pathological stage N.

**Figure 2:**
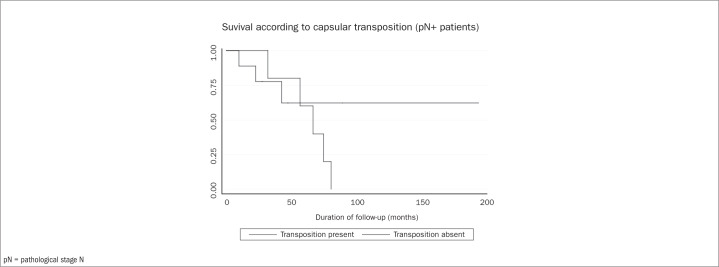
Overall survival rates for patients with extracapsular spread.

**Figure 3:**
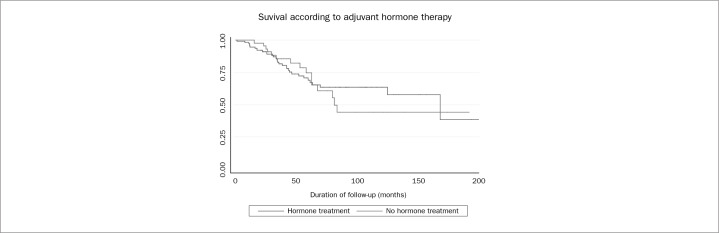
Overall survival rates for patients with and without postoperative hormone therapy.

After grouping the variables in steps, multivariate analysis was performed. Table 1 details each step and shows which variables were evaluated and eliminated over the course of the analysis. After the final step, presence of metastatic lymph nodes (Figure 1), capsular transposition (Figure 2), lymph node ratio and hormone therapy (Figure 3) were found to be significant in the multivariate analysis.

**Table 1.** Multivariate analysis on prognostic factors

Chunk steps	Features	P value	Hazard ratio	95% confidence interval
I	Clinical stage T (T1/2 versus T3/4)	0.070	1.815	0.952-3.461
	Clinical stage N (N-/N+)	0.109	1.693	0.889-3.224
	Tumor size	0.002	2.876	1.456-5.679
II	Tumor size	0.675	1.210	0.496-2.952
	Skin invasion	0.361	1.388	0.687-2.804
	Pathological stage T (T1/2 versus T3/4)	0.000	5.211	2.093-12.970
III	Pathological stage T (T1/2 versus T3/4)	0.002	3.125	1.505-6.488
	Vascular invasion	0.016	2.384	1.178-4.821
	Pathological stage N (N-/N+)	0.000	6.932	2.467-19.483
IV	Pathological stage T (T1/2 versus T3/4)	0.002	3.130	1.509-6.496
	Pathological stage N (N-/N+)	0.001	7.547	2.410-23.634
	Vascular invasion	0.016	2.475	1.184-5.175
	Lymphatic invasion	0.722	0.825	0.285-2.385
V	Pathological stage T (T1/2 versus T3/4)	0.004	2.985	1.412-6.311
	Pathological stage N (N-/N+)	0.000	6.998	2.494-19.639
	Vascular invasion	0.104	2.059	0.862-4.915
	Neural invasion	0.582	1.279	0.533-3.068
VI	Pathological stage T (T1/2 versus T3/4)	0.000	3.498	1.779-6.878
	Pathological stage N (N-/N+)	0.000	7.781	3.124-19.378
	Estrogen receptor presence	0.400	0.718	0.332-1.554
VII	Pathological stage T (T1/2 versus T3/4)	0.075	3.417	0.884-13.212
	Pathological stage N (N-/N+)	0.020	12.431	1.491-103.675
	Progesterone receptor status	0.934	0.956	0.334-2.740
VIII	Pathological stage N (N-/N+)	0.007	20.411	2.272-183.366
	Lymph node capsular transposition	0.011	2.938	1.285-6.719
IX	Pathological stage N (N- / N+)	0.000	14.023	5.737-34.278
	Lymph node capsular transposition	0.013	2.877	1.248-6.635
	Postoperative chemotherapy	0.214	1.497	0.792-2.827
X	Pathological stage N (N-/N+)	0.000	12.127	5.082-28.934
	Lymph node capsular transposition	0.001	3.490	1.718-7.091
	Hormone therapy	0.041	1.920	1.005-3.704
	Lymph node ratio	0.002	1.874	1.232-2.935

## DISCUSSION

In this report, we analyzed the prognostic factors for ILC in a manner not performed before in the literature, with multivariate analysis performed through chunkwise testing methods with a backward elimination procedure. It allowed us to diminish the number of variables analyzed at each step. One major limitation of this study was its retrospective nature, but lobular carcinomas of the breast are not as common as ductal carcinomas and prospective studies are difficult to conduct because of the length of time involved in amassing a significant number of patients.

The prognostic factors for ILC have already been assessed in other reports. A previous study analyzed 390 patients and demonstrated that age (P = 0.0039), stage T (T1/2 versus T3, P = 0.0099), lymph node status (N0/1 versus N2, P = 0.0009), and grade (I/II versus III, P = 0.0128) affected the prognosis, in a multivariate model.^10^ The role of lymph node status, capsular transposition and the size of the metastatic deposits have also been demonstrated in other reports.^11^ Histological grading has also been shown to be a significant prognostic factor. In a report from the Danish Breast Cancer Cooperative Group, patients with grade III tumors showed a higher proportion of non-classical subtypes and significantly worse prognosis for overall and disease-free survival, in comparison with patients with grade I/II carcinomas.^12^ In another report, histological grading was correlated with other factors for poor prognosis, such as increasing tumor size, positive lymph nodes, vascular invasion and estrogen receptor negativity. Multivariate analysis has shown that histological grading is a significant factor indicating shorter disease-specific and disease-free survival.^13^ The role of bilateral disease as a prognostic factor has also been assessed. In a previous report, it was shown that patients with synchronous, bilateral ILC had a significant worse prognosis than did patients with unilateral or metachronous bilateral disease (P < 0.02).^14^

Information on prognostic factors for ILC may be also found in reports that had the original intent of comparing this histological type with IDC. In a report originally intended to compare the prognostic significance of lobular histology, the following factors were found to be significantly linked to overall survival in cases of ILC: node status, age, primary tumor size, ER status and S-phase.^5^ Another study also compared the prognosis using histological analysis and concluded that for ILC, the following factors were significant in multivariate analysis: age at diagnosis, tumor size, lymph node status and pathological stage. In another report analyzing the prognostic factors for 217 patients with ILC, the following factors were found to be significant: tumor size (P < 0.0001), axillary nodal metastasis (P < 0.0001), absence of tumor necrosis (P < 0.0001), low mitotic count (P < 0.0001), low histological grading (P = 0.001) and S-phase fraction lower than the median (P = 0.005).^15^ Age at diagnosis, tumor size, pathological stage and lymph node status were shown to be independent prognostic indicators for 10-year survival, with no significant prognostic difference between ILC and IDC.^16^ The lymph node ratio has been shown to be an important predictor for survival among patients with stage I and II breast cancer.^17^

In our analysis, the number of factors found to be significant was smaller than in these other series. Age at diagnosis was found to be nonsignificant at different cutoff points and as a continuous variable, and tumor size was only significant in the univariate analysis, and thus was excluded from the multivariate analysis. No significance was found in the multivariate analysis for ER status or histological grading, which were significant in previous reports.^5^ On the other hand, postoperative hormone therapy was significant for survival.

## CONCLUSIONS

From the data in this report, we conclude that the most important prognostic factors for lobular carcinoma of the breast seem to relate to lymph node status and presence of capsular transposition. The presence of estrogen receptors and the inclusion of postoperative hormone therapy was also associated with a significant improvement in survival. However, further prospective studies would be needed to confirm this evidence.

## References

[1] Berg JW, Hutter RV (1995). Breast cancer. Cancer.

[2] Li CI, Anderson BO, Daling JR, Moe RE (2003). Trends in incidence rates of invasive lobular and ductal breast carcinoma. JAMA.

[3] Fisher ER, Gregorio RM, Fisher B (1975). The pathology of invasive breast cancer. A syllabus derived from findings of the National Surgical Adjuvant Breast Project (protocol no. 4). Cancer.

[4] Martinez V, Azzopardi JG (1979). Invasive lobular carcinoma of the breast: incidence and variants. Histopathology.

[5] Arpino G, Bardou VJ, Clark GM, Elledge RM (2004). Infiltrating lobular carcinoma of the breast: tumor characteristics and clinical outcome. Breast Cancer Res.

[6] Ferlicot S, Vincent-Salomon A, Médioni J (2004). Wide metastatic spreading in infiltrating lobular carcinoma of the breast. Eur J Cancer.

[7] Cocquyt VF, Schelfhout VR, Blondeel PN (2003). The role of biological markers as predictors of response to preoperative chemotherapy in large primary breast cancer. Med Oncol.

[8] Greene FL, Compton CC, Fritz AG, Shah J, Winchester DP (2002). AJCC Cancer staging atlas.

[9] Kleinbaum DG, Kupper LL, Muller KE, Nizam A (1998). Applied regression analysis and multivariate methods.

[10] Moreno-Elola A, Aguilar A, Roman JM (1999). Prognostic factors in invasive lobular carcinoma of the breast: a multivariate analysis. A multicentre study after seventeen years of follow-up. Ann Chir Gynaecol.

[11] Ladekarl M, Sørensen FB (1993). Prognostic, quantitative histopathologic variables in lobular carcinoma of the breast. Cancer.

[12] Talman ML, Jensen MB, Rank F (2007). Invasive lobular breast cancer. Prognostic significance of histological malignancy grading. Acta Oncol.

[13] Rakha EA, El-Sayed ME, Menon S, Green AR, Lee AH, Ellis IO (2008). Histologic grading is an independent prognostic factor in invasive lobular carcinoma of the breast. Breast Cancer Res Treat.

[14] Dixon JM, Anderson TJ, Page DL, Lee D, Duffy SW, Stewart HJ (1983). Infiltrating lobular carcinoma of the breast: an evaluation of the incidence and consequence of bilateral disease. Br J Surg.

[15] Toikkanen S, Pylkkänen L, Joensuu H (1997). Invasive lobular carcinoma of the breast has better short- and long-term survival than invasive ductal carcinoma. Br J Cancer.

[16] Jayasinghe UW, Bilous AM, Boyages J (2007). Is survival from infiltrating lobular carcinoma of the beast different from that of infiltrating ductal carcinoma?. Breast J.

[17] van der Wal BC, Butzelaar RM, van der Meij S, Boermeester MA (2002). Axillary lymph node ratio and total number of removed lymph nodes: predictors of survival in stage I and II breast cancer. Eur J Surg Oncol.

